# Draft Genome Sequence of Enterobacter hormaechei ENT5, a Component of the Symbiotic Community of Tephritid Flies

**DOI:** 10.1128/MRA.01364-19

**Published:** 2019-12-05

**Authors:** Elias Asimakis, Vangelis Doudoumis, Georgia Gouvi, George Tsiamis

**Affiliations:** a Department of Environmental Engineering, University of Patras, Agrinio, Greece; University of Maryland School of Medicine

## Abstract

Enterobacter strains are among the dominant symbiotic bacteria in the gastrointestinal tract of insects, with the ability to fulfill diverse roles. In this announcement, we describe the draft genome sequence of Enterobacter hormaechei strain ENT5, isolated from wild adult Zeugodacus cucurbitae flies.

## ANNOUNCEMENT

Symbiotic bacterial communities associated with insects are continuously drawing attention as a means to improve environmentally friendly techniques, such as the sterile insect technique (SIT), for the effective control of insect pest populations (1). Many fruit flies, members of the family Tephritidae, are good examples of persistent, destructive agricultural pests with the ability to affect a variety of cultivated plants worldwide (2–5).

Enterobacter spp. are Gram-negative, rod-shaped, facultatively anaerobic bacteria that are components of the gastrointestinal symbiotic communities of insects (6–9) and are responsible for the provision of nutrients (10, 11), pathogen transmission (12), communication (13), and interactions with the host plant (14). Another interesting aspect of this association is the probiotic effect that many *Enterobacter* strains exhibit, which results in the enhancement of important biological parameters of many mass-reared fly species, including Zeugodacus cucurbitae (15–17).

In this article, we report the draft genome sequence of Enterobacter hormaechei ENT5, isolated from wild *Z. cucurbitae* flies that were collected in Mauritius in 2014. Flies were surface sterilized with 70% ethanol and washed with a sterile 1% phosphate-buffered saline (PBS) solution. Three male and three female adult flies were pooled and homogenized in the sterile 1% PBS solution. The homogenate was serially diluted, and 100 μl was plated on Luria-Bertani (LB) medium (1% [wt/vol] peptone, 1% [wt/vol] NaCl, 0.5% [wt/vol] yeast extract, and 1.5% [wt/vol] agar) and incubated at 25°C under aerobic conditions. The *E. hormaechei* strain was identified among the isolates, and its genomic DNA was extracted from a single colony using lysis buffer containing lysozyme, according to Haught et al. (18). The DNA was sent to MicrobesNG (Birmingham, UK) for whole-genome sequencing. DNA was quantified using the Quant-iT double-stranded DNA (dsDNA) high-sensitivity (HS) assay (Invitrogen) in an Eppendorf AF2200 plate reader. A genomic DNA library was prepared using the Nextera XT library prep kit (Illumina, San Diego, CA, USA), following the manufacturer’s protocol. The library was quantified using the Kapa Biosystems library quantification kit for Illumina on a Roche LightCycler 96 quantitative PCR (qPCR) machine and sequenced with 75-fold coverage on the Illumina HiSeq 2500 using a 250-bp paired-end protocol, producing 1,243,783 reads. Reads were trimmed using Trimmomatic v.0.3, with a sliding window quality cutoff of Q15 (19), and their quality was assessed using FastQC 0.11 (20). Default program parameters were used. Reads were *de novo* assembled into 446 contigs using SPAdes 3.7 (21). The quality of the assembly was evaluated with QUAST 5.0.2 (22). Taxonomic assignment of reads was performed with Kraken 2.0.7 (23), and genome annotation was performed using Prokka 1.11 (24). Protein function was predicted by performing sequence similarity queries against UniProtKB (25).

The assembled draft genome was 4,774,740 bp long, with a GC content of 55.93%. The *N*_50_ was 201,158 bp, the *L*_50_ was 8, and the largest contig was 675,820 bp long. Almost half of the reads (41.4%) were classified within the genus *Enterobacter*, while 42.92% remained unclassified, and the reads showed 98.9% similarity based on average nucleotide identity (ANI) to those of the type genome of Enterobacter hormaechei. The draft genome contained 4,344 protein-coding sequences, 968 of which were identified as hypothetical proteins. Sequences coding for 81 tRNA genes, 8 5S rRNA genes, and 1 16S rRNA gene were also identified. Genome annotation revealed the genes *nfsA* and *nfsB,* which code for oxygen-insensitive NAD(P)H nitroreductases responsible for nitrogen fixation.

### Data availability.

The whole-genome shotgun project of *E. hormaechei* ENT5 has been deposited at DDBJ/ENA/GenBank under the accession number VTSZ00000000, BioProject number PRJNA562639, and BioSample number SAMN12646888. Raw sequencing reads were deposited to the Sequence Read Archive under accession number SRR10028728. The version described in this paper is version VTSZ01000000.
